# Data on the identification and characterization of by-products from *N*-Cbz-3-aminopropanal and *t*-BuOOH/H_2_O_2_ chemical reaction in chloroperoxidase-catalyzed oxidations

**DOI:** 10.1016/j.dib.2016.06.028

**Published:** 2016-06-23

**Authors:** Gerard Masdeu, Míriam Pérez-Trujillo, Josep López-Santín, Gregorio Álvaro

**Affiliations:** a Bioprocess Engineering and Applied Biocatalysis Research Group, Departament d’Enginyeria Química, Biològica i Ambiental, Universitat Autònoma de Barcelona, 08193 Bellaterra, Catalonia, Spain; b Servei de Ressonància Magnètica Nuclear, Universitat Autònoma de Barcelona, 08193 Bellaterra, Catalonia, Spain

**Keywords:** *N*-Cbz-3-aminopropanal, Aldehyde-peroxide reaction, NMR identification

## Abstract

This data article is related to the subject of a publication in Process Biochemistry, entitled “Chloroperoxidase-catalyzed amino alcohol oxidation: Substrate specificity and novel strategy for the synthesis of *N*-Cbz-3-aminopropanal” (Masdeu et al., 2016) [1]. Here, the products of the chemical reaction involving the amino aldehyde (*N*-Cbz-3-aminopropanal) and peroxides (*tert*-butyl hydroperoxide and H_2_O_2_) are characterized by NMR. ^1^H and ^13^C NMR full characterization of the products was obtained based on 2D NMR, 1D selective NOESY and diffusion spectroscopy (DOSY) experiments.

Specifications Table

**Table:** 

Subject area	*Biotechnology, Biochemistry*
More specific subject area	*Biocatalysis*
Type of data	*Figures, scheme, table*
How data was acquired	*NMR data adquired on a Bruker Avance II 600 nuclear magnetic resonance spectrometer (Bruker Biospin) equipped with a 5 mm TBI probe with Z-gradients and a TCU (temperature control unit).*
Data format	*Analyzed*
Experimental factors	*By-products were obtained by 24-h incubation of the amino aldehyde and peroxides.*
Experimental features	*Dried samples were dissolved in CDCl*_*3*_*and/or D*_*2*_*O and analyzed.*^*1*^*H (600.13 MHz) and*^*13*^*C (150.13 MHz) NMR spectra were recorded at 298.0 K of temperature. Spectra of CDCl*_*3*_*samples were calibrated using the residual solvent signal (7.26 ppm for*^*1*^*H and 77.16 for*^*13*^*C) and spectra of aqueous samples using TSP as internal reference.*
Data source location	*Bellaterra, Spain, Universitat Autònoma de Barcelona*
Data accessibility	*Data is with this article.*

**Value of the data**

• NMR-based concerted analysis is helpful for the identification of complex molecules by NMR correlations.
• A simple ^1^H NMR experiment and comparison of signal chemical shifts and multiplicities are beneficial for the rapid identification of the products from a chemical reaction aldehyde-peroxide.
• Data in ^1^H NMR and ^13^C NMR is valuable for related chemical reactions between peroxides and an aldehyde.

## 1 Data

This paper deals about the identification of the products from the chemical reaction between *N-*Cbz-3-aminopropanal (β-CHO) and *tert*-butyl hydroperoxide (*t*-BuOOH) or H_2_O_2_. It describes the preparation of the samples prior the NMR measurements, and the concerted analysis of the NMR spectra and 2D correlations.

## 2 Experimental design, materials and methods

**Scheme 1:**
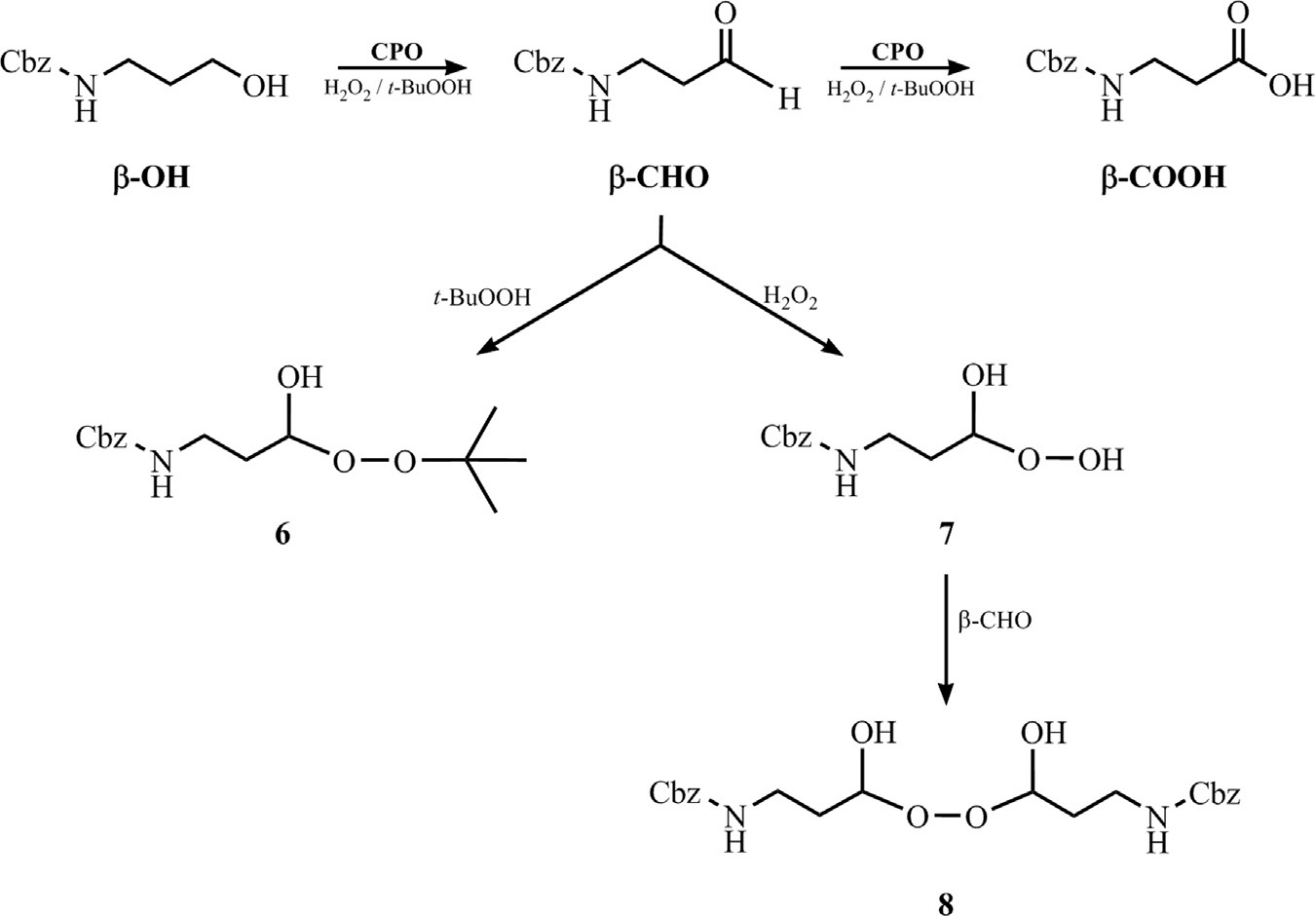
Reaction scheme extracted from the related research article [1]. The formed amino aldehyde from the chloroperoxidase-catalyzed *N*-Cbz-3-aminopropanol (β-OH) oxidation reacts with either *t*-BuOOH or H_2_O_2_. Compounds **6**–**8** have been characterized by NMR data.

Three preparative reactions were carried out in order to obtain enough amounts of by-products (compounds **6**–**8**, Scheme 1) for further analyses. β-CHO (17 mM, maximum solubility) was dissolved in 10 mL water. Selected peroxide was added to the reaction medium and left in incubation for 24 h. For compound **6** preparation, 250 mM *t-*BuOOH was employed (ca 50% yield); for **7**, 600 mM H_2_O_2_ (ca 65% yield); for **8**, 72 mM H_2_O_2_ (ca 99% yield). All reactions were performed at 25 °C, 1000 rpm of MultiTherm™ orbital stirring. Compounds **6–7** were carefully filtered prior the analysis to eliminate impurities. Compound **8** was isolated by filtration and cautiously dried at 35 °C.

For the identification of the product from reaction between β-CHO and *t*-BuOOH, the reaction medium (containing product **6**) was directly analyzed. 200 µL of D_2_O (99.96% D), containing 0.3% of TSP (trimethylsilyl propanoic acid), were added to a 400 µL aliquot of the aqueous crude and the dissolution was transferred to a 5-mm-diameter NMR tube. To analyze the reaction intermediate in the β-CHO-H_2_O_2_ reaction (compound **7**), 200 µL of D_2_O (99.96% D), containing 0.3% of TSP, were added to a 400 µL aliquot of the aqueous sample of the reaction crude. The solution was transferred to a 5-mm-diameter NMR tube. For compound **8** identification, the dried reaction by-product (20.2 mg) of the oxidation reaction between β-CHO and H_2_O_2_ was dissolved in 600 µL of CDCl_3_ (99.96% D).

^1^H (600.13 MHz) and ^13^C (150.13 MHz) NMR spectra were recorded at 298.0 K of temperature on a Bruker Avance II 600 nuclear magnetic resonance spectrometer (Bruker Biospin, Rheinstetten, Germany) equipped with a 5 mm TBI probe with Z-gradients and a TCU (temperature control unit). Initially, 1D ^1^H NMR spectra of all samples were acquired. For that, a standard 90° pulse sequence, with an acquisition time of 1.71 s and a relaxation delay of 2 s was recorded. Data were collected into 32 K computer data points, with a spectral width of 9590 Hz and as the sum of 1024 transients. The resulting free induction decays (FIDs) were Fourier transformed manually phased and baseline corrected. In the case of samples containing H_2_O, the peak of the protonated water was suppressed by the standard presaturation of the signal.

The structural characterization of compounds was carried out with the aid of 2D NMR experiments, such as COSY (Correlated Spectroscopy), HSQC (Heteronuclear Single Quantum Correlation), HMBC (Heteronuclear Multiple Bond Correlation), NOESY (Nuclear Overhauser and Exchange Spectroscopy), DOSY (Diffusion Spectroscopy) and 1D selective NOESY experiments performed under standard conditions. When required, solvent suppression techniques were applied. Spectra of CDCl_3_ samples were calibrated using the residual solvent signal (7.26 ppm for ^1^H and 77.16 for ^13^C) and spectra of aqueous samples using TSP as internal reference.

**Fig. 1:**
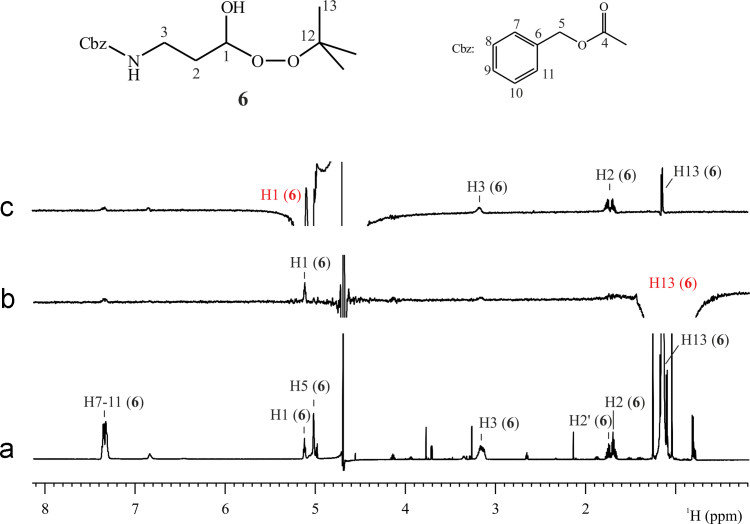
NMR spectra of the reaction medium of β-CHO and *t-*BuOOH oxidation with solvent H_2_O–D_2_O (67:33). (a) ^1^H NMR spectrum of the sample with suppression of the H_2_O signal, (b) ^1^H 1D selective NOESY spectrum with irradiation of H13 signal at 1.15 ppm, and (c) ^1^H 1D selective NOESY spectrum with irradiation of H1 signal at 5.13 ppm. Experiments acquired at 298.0 K and at a magnetic field of 600 MHz.

The NMR analysis of the reaction media of β-CHO and *t-*BuOOH oxidation, revealed the formation of compound **6**. The results obtained from 1D ^1^H, COSY and 1D selective NOESY experiments of the aqueous media of reaction, allowed the ^1^H NMR characterization of the molecule. Fig. 1 shows the structure of molecule **6** and the ^1^H spectrum of the reaction media with the assignment of proton signals of **6**. Fig. 1b and c shows, respectively, the 1D selective NOESY spectra obtained when signals in the *t-*butyl region (1.15 ppm) and when signal corresponding to H1 (5.13 ppm) was irradiated. NOESY correlation between H1 and H13 confirmed the presence of the *t-*butyl moiety in the molecule (see Table 1).

**Fig. 2:**
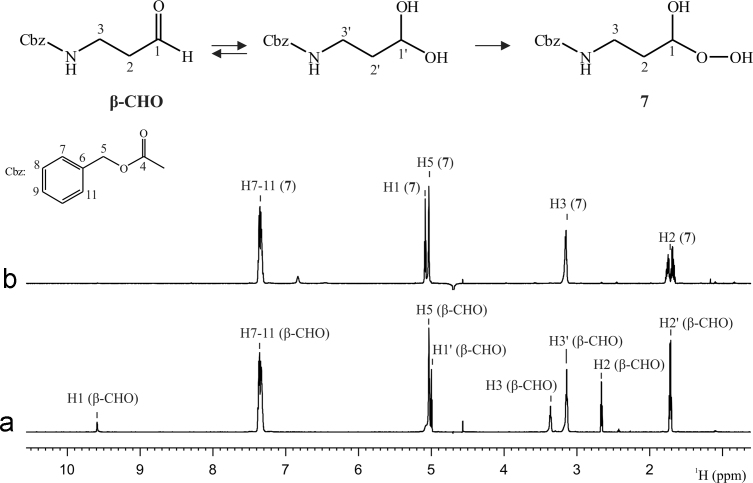
^1^H NMR spectrum of (a) β-CHO in D_2_O and of (b) an aliquot of the β-CHO-H_2_O_2_ reaction media in H_2_O–D_2_O (67:33). Experiments acquired at 298.0 K and at a magnetic field of 600 MHz.

The intermediate of the reaction β-CHO-H_2_O_2_ was identified as the hydroxy peroxy derivative of β-CHO, compound **7**. The ^1^H NMR spectra of β-CHO dissolved in D_2_O and of an aliquot from the mentioned chemical reaction in H_2_O–D_2_O (67:33) were compared. In the case of β-CHO, two species were observed in the aqueous solution, which corresponded to the equilibria of the aldehydic and the acetal forms of the molecule (see Fig. 2a). Some important differences were observed between the β-CHO spectra and that of the reaction sample in aqueous media. In the case of the later, just one species was observed, meaning that the aldehydic and/or acetal forms were not present anymore. Besides, the signal corresponding to H1 was slightly down field shifted compared to the acetal form of β-CHO (from 5.00 to 5.09 ppm), which was consistent with the presence of a hydroxy peroxy moiety (see Table 1). Also, no other unidentified signals were observed in the ^1^H spectrum (see Fig. 2b).

**Fig. 3:**
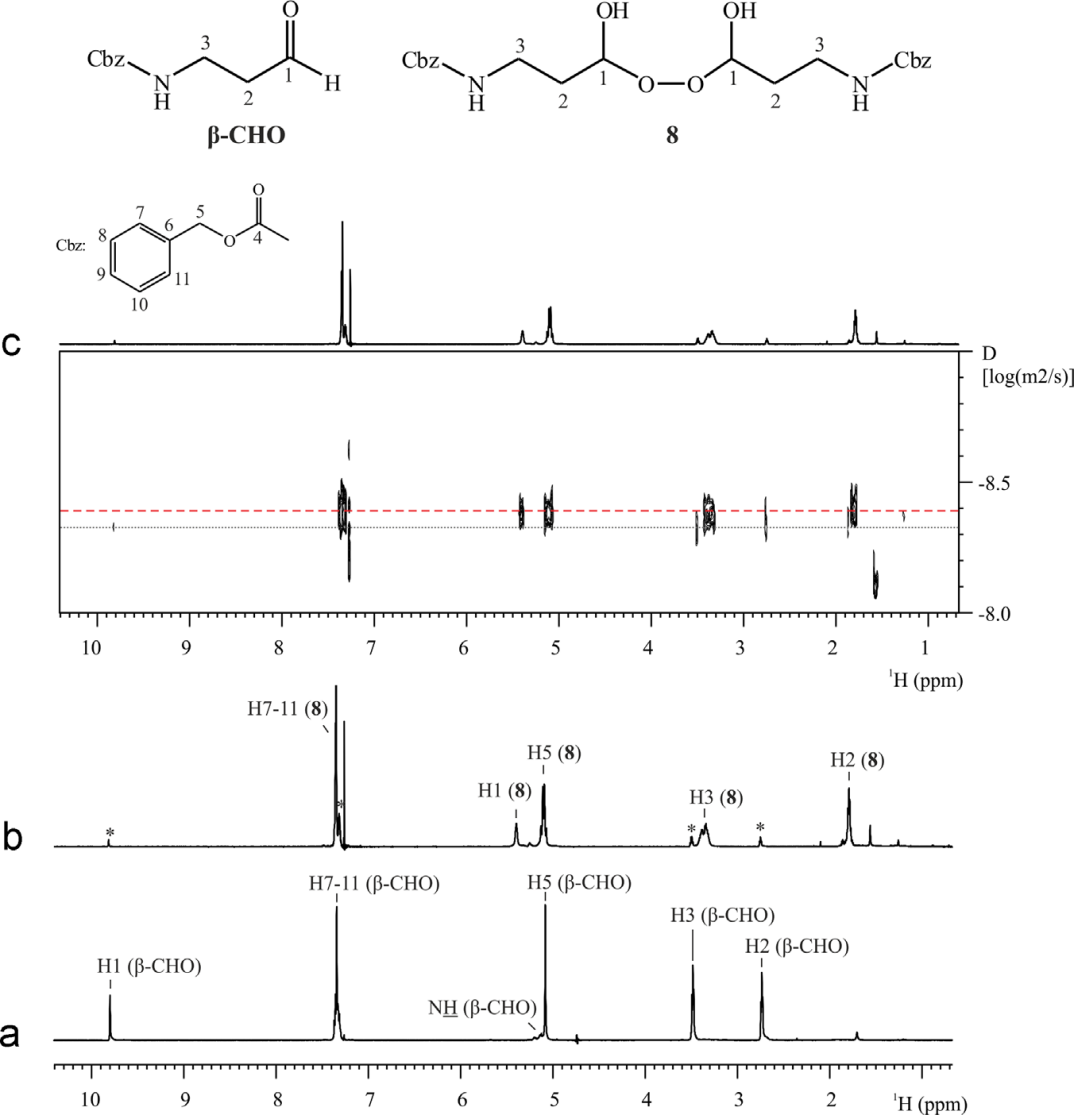
NMR spectra of β-CHO and **8** in CDCl_3_. (a) ^1^H NMR spectrum of β-CHO, (b) ^1^H NMR spectrum and c) ^1^H DOSY spectrum of sample containing compound **8** and reactive β-CHO (indicated with *). Experiments acquired at 298.0 K and at a magnetic field of 600 MHz.

NMR spectroscopy allowed the identification of compound **8** yielded by the oxidation of β-CHO with hydrogen peroxide (see Fig. 3). Initially, β-CHO was ^1^H and ^13^C fully characterized by the concerted analysis of the 2D NMR correlations COSY, HSQC and HMBC (see Fig. 3 and Table 1) [2], [3] Likewise, a second sample consisting in the product of the oxidation reaction, dissolved in CDCl_3_, was analyzed. The analysis showed a new molecule, **8**, and the presence of reactive β-CHO in a smaller amount. Fig. 3 shows the structure of **8** and the ^1^H NMR spectra of the analyzed samples with the assignment of the signals. Briefly, comparing with the spectrum of β-CHO, compound **8** showed no aldehydic proton and a new signal appeared at *δ*(^1^H) 5.39 ppm and *δ*(^13^C) 99.37 ppm. Also, protons H2 and H3 resonated at lower frequencies regarding their analogues of β-CHO (see Fig. 3). The DOSY experiment performed in the second sample (see Fig. 3c and Table 1) showed a significant lower diffusion (i.e. a smaller diffusion coefficient, D) of molecule **8** respect to β-CHO. This result, together with the information yielded by the 2D correlations, confirmed the dimeric structure of compound **8**.

Products **6**–**8** masses were confirmed by mass spectrometry (protocol not detailed, Supplementary material): HPLC-MS-MS (1200RR LC – Agilent Technologies, Santa Clara, CA, USA – and micrOTOF-Q with Apollo II Electrospray ion source – Bruker Technologies, Billerica, MA, USA) or MS (micrOTOF-Q II with Apollo II Electrospray ion source – Bruker Technologies). Those analyses were executed by SAQ (Servei d’Anàlisi Química, UAB, Barcelona, Spain).

### 2.1 Complete characterization of the three compounds is detailed below

**Benzyl (3-(tert-butylperoxy)-3-hydroxypropyl)carbamate (6):** Colorless; ^1^H NMR (600 MHz, H_2_O–D_2_O 67:33): *δ*=7.39–7.29 (br m, 5H), 5.13 (t, *J*=5.8, 1H), 5.03 (br s, 2H), 3.17 (br m, 2H), 1.75 (m, 1H), 1.69 (m, 1H), 1.15 (s, 9H); MS-ESI+: *m*/*z*=320.1469, calcd. for C_15_H_23_NO_5_: 320.1468.

**Benzyl (3-hydroperoxy-3-hydroxypropyl)carbamate (7):** Colorless; ^1^H NMR (600 MHz, H_2_O–D_2_O 67:33): *δ*=7.39–7.29 (br m, 5H), 5.09 (t, *J*=5.8, 1H), 5.04 (br s, 2H), 3.17 (br m, 2H), 1.74 (m, 1H), 1.69 (m, 1H); ^13^C NMR (150 MHz, CDCl_3_): *δ*=158.1, 136.4, 128.9, 128.5, 128.3, 99.3, 66.8, 36.5, 32.4; HPLC-MS-ESI+: *m*/*z*=264.0856, calcd. for C_11_H_15_NO_5_: 264.0842.

**Dibenzyl (peroxybis(3-hydroxypropane-3,1-diyl))dicarbamate (8):** White solid; ^1^H NMR (600 MHz, CDCl_3_): δ=7.39–7.29 (br m, 10H), 5.39 (br m, 2H), 5.09 (br m, 4H), 3.36 (br m, 4H), 1.79 (br m, 4H); ^13^C NMR (150 MHz, CDCl_3_): *δ*=157.0, 136.2, 128.9, 128.5, 128.3, 99.4, 66.7, 36.1, 33.6; MS-ESI+: *m*/*z*=471.1744, calcd. for C_22_H_28_N_2_O_8_: 471.1738.

## Figures and Tables

**Table 1:** ^1^H and ^13^C NMR chemical shifts (*δ*) and multiplicity of β-CHO and compounds **6**–**8** at 298.0 K of temperature.

	**β-CHO**^a^		**6**^b^	**7**^b^		**8**^a^	
	*δ* (^1^H)	*δ* (^13^C)	*δ* (^1^H)	*δ* (^1^H)	*δ* (^13^C)	*δ* (^1^H)	*δ* (^13^C)
Id.	[ppm]	[ppm]	[ppm]	[ppm]	[ppm]	[ppm]	[ppm]
1	9.79 (s)^c^	201.0	5.13 (t)	5.09 (t)	99.3	5.39 (m)	99.4
2	2.73 (t)	43.9	1.69 (m)/1.75 (m)	1.69 (m)/1.74 (m)	32.4	1.79 (m)	33.6
3	3.48 (t)	34.2	3.17 (m)	3.17 (m)	36.5	3.36 (m)	36.1
4	–	156.5	–	–	158.1	–	157.0
5	5.08 (s, br)	66.7	5.03 (s, br)	5.04 (s, br)	66.8	5.09 (s, br)	66.7
6	–	136.7	–	–	136.4	–	136.2
7-11	7.29–7.39 (m, br)	126.2–130.3	7.29–7.39 (m, br)	7.29–7.39 (m, br)	126.2–130.3	7.29–7.39 (m, br)	126.2–130.3
13	–	–	1.15 (s)	–	–	–	–

a In CDCl_3_.

b In H_2_O–D_2_O (67:33).

c Signal multiplicity: s, singlet; t, triplet; m, multiplet; br, broad.
