# Supplementary material for: Data on the identification and characterization of by-products from N-Cbz-3-aminopropanal and t-BuOOH/H2O2 chemical reaction in chloroperoxidase-catalyzed oxidations
Source: Data Brief. 2016 Jun 23;8:659–65. doi: 10.1016/j.dib.2016.06.028 (PMC4939320; doi:10.1016/j.dib.2016.06.028)
Supplement: Supplementary file 1 — Supplementary material [file mmc1.docx]

*Conflict of Interest Data article*

**Title:** Data on the identification and characterization of by-products from *N*-Cbz-3-aminopropanal and *t*-BuOOH/H_2_O_2_ chemical reaction in chloroperoxidase-catalyzed oxidations

**Authors:** Gerard Masdeu^a,^*, Míriam Pérez-Trujillo^b,^*, Josep López-Santín^a,^**, Gregorio Álvaro^a^

Conflict of interests: None

Signed: Josep López-Santín

Corresponding author

On behalf of all authors
