# Supplementary material for: Data on the identification and characterization of by-products from N-Cbz-3-aminopropanal and t-BuOOH/H2O2 chemical reaction in chloroperoxidase-catalyzed oxidations
Source: Data Brief. 2016 Jun 23;8:659–65. doi: 10.1016/j.dib.2016.06.028 (PMC4939320; doi:10.1016/j.dib.2016.06.028)
Supplement: Supplementary file 2 — Supplementary material [file mmc2.docx]

**Supplementary material**

Data on the identification and characterization of by-products from *N*-Cbz-3-aminopropanal and *t*-BuOOH/H_2_O_2_ chemical reaction in chloroperoxidase-catalyzed oxidations

Gerard Masdeu,^a,^* Míriam Pérez-Trujillo,^b,^* Josep López-Santín,^a,^** and Gregorio Álvaro^a^

^a^ Bioprocess Engineering and Applied Biocatalysis Group. Departament d’Enginyeria Química, Biològica i Ambiental. Universitat Autònoma de Barcelona, 08193 Bellaterra, Catalonia, Spain

^b^ Servei de Ressonància Magnètica Nuclear, Universitat Autònoma de Barcelona, 08193 Bellaterra, Catalonia, Spain

*Both authors contributed equally to this work.

**Corresponding author:

Josep López-Santín

Bioprocess Engineering and Applied Biocatalysis Group. Departament d’Enginyeria Química, Biològica i Ambiental. Universitat Autònoma de Barcelona, 08193 Bellaterra, Catalonia, Spain

Tel.: +34 93 581 1018

Fax: +34 93 581 2013

E-mail: josep.lopez@uab.cat

**MS analysis**


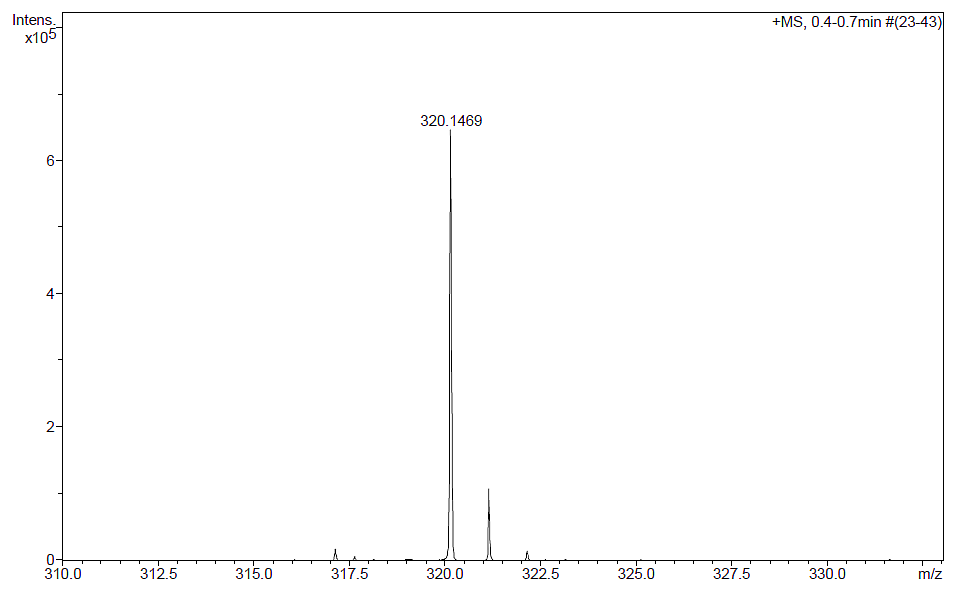


**Figure S1.** MS–ESI+ spectra of the identified compound **6** (*m*/*z* = 320.1469), obtained by the chemical reaction between *N-*Cbz-3-aminopropanal and *t-*BuOOH.


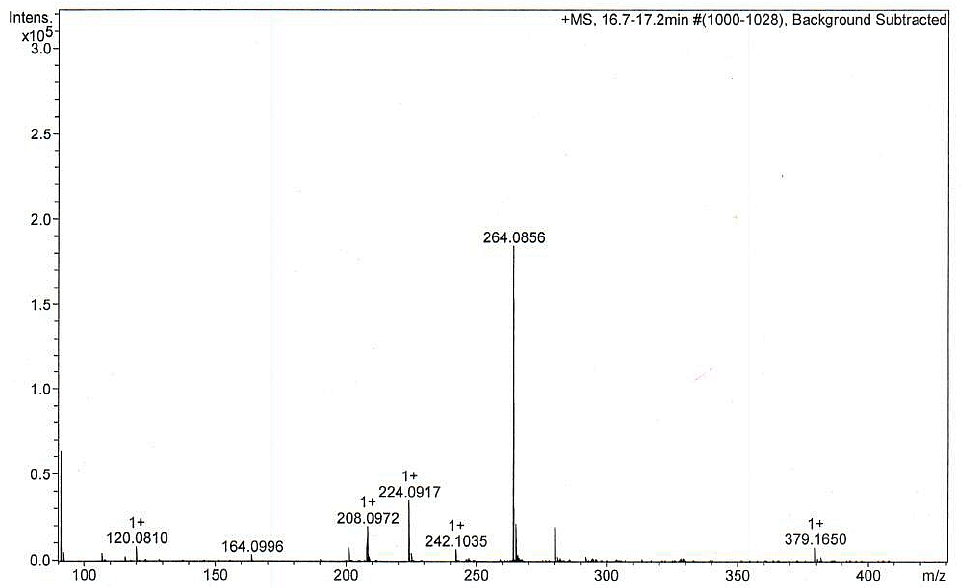


**Figure S2.** HPLC–MS–ESI+ spectra of the identified compound **7** (*m*/*z* = 264.0856), obtained by the chemical reaction between *N-*Cbz-3-aminopropanal and H_2_O_2_.


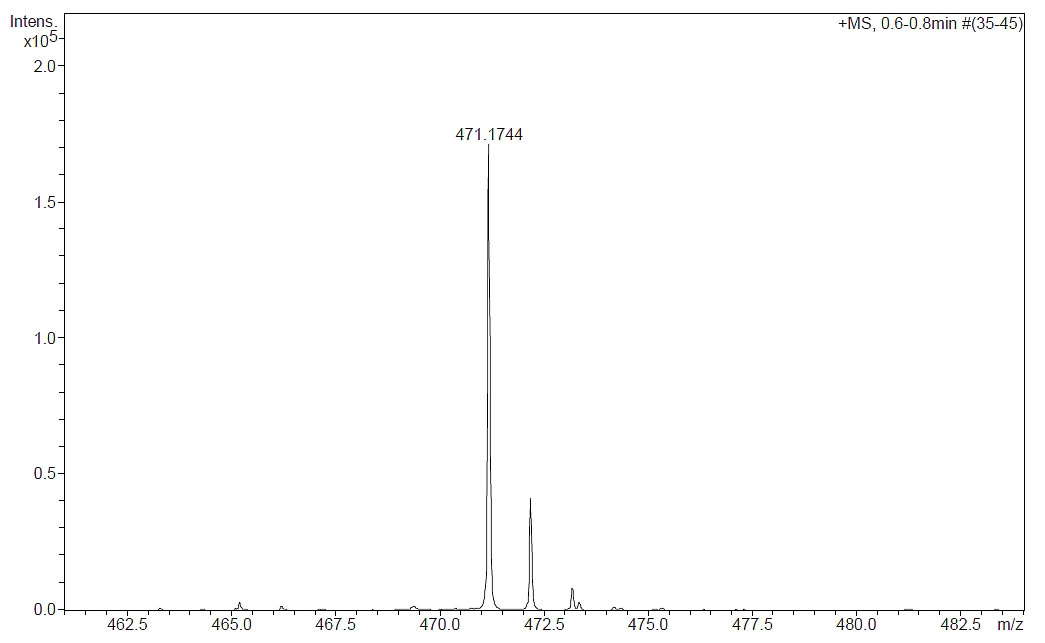


**Figure S3.** MS–ESI+ spectra of the identified compound **8** (*m*/*z* = 471.1744), obtained by further chemical reaction between *N-*Cbz-3-aminopropanal and the product from the reaction of *N-*Cbz-3-aminopropanal and *t-*BuOOH.
